# Oxidative Stress, Environmental Pollutants, Aging, and Epigenetic Regulation: Mechanistic Insights and Biomarker Advances

**DOI:** 10.3390/antiox15040494

**Published:** 2026-04-16

**Authors:** Minelly Krystal Gonzalez Acevedo, Michael Powers, Luca Cucullo

**Affiliations:** 1 Department of Biological and Biomedical Sciences, Oakland University, Rochester, MI 48309, USA; mkgonzalez@oakland.edu (M.K.G.A.); michaelpowers@oakland.edu (M.P.); 2 Department of Foundation Medical Studies, Oakland University William Beaumont School of Medicine, 586 Pioneer Dr, Rochester, MI 48309, USA

**Keywords:** environmental pollutants, oxidative stress, redox signaling, mitochondria, NADPH oxidase, epigenetics, DNA methylation, histone modifications, non-coding RNA, biological aging, biomarkers, exposomics

## Abstract

Environmental pollutants, lifestyle factors, and intrinsic metabolism can amplify reactive oxygen and nitrogen species (ROS/RNS) generation beyond antioxidant capacity. The resulting oxidative stress damages macromolecules, perturbs redox signaling, and may accelerate biological aging. This review synthesizes evidence published mainly in 2020–2025 on how major pollutant classes (air pollutants, metals, pesticides, nanoparticles, and micro-/nanoplastics) induce ROS through shared nodes mitochondrial electron transport disruption, NADPH oxidase activation, and redox cycling/Fenton chemistry and how these signals propagate to epigenetic remodeling (DNA methylation, histone modifications, and non-coding RNAs). To move beyond descriptive cataloging, we grade the strength of evidence by study context (cell culture, animal models, human observational studies, and clinically oriented biomarker research), highlight convergent findings and unresolved controversies, and specify key methodological limits. We then compare oxidative-stress biomarker platforms by analytical specificity, pre-analytical susceptibility, and translational readiness, distinguishing validated markers from exploratory redox-epigenetic and multi-omics signatures. Finally, we discuss how exposomics and AI-assisted multi-omics integration may support biomarker discovery while emphasizing current constraints (confounding, batch effects, and limited prospective validation) that must be addressed for clinical translation.

## 1. Introduction

Oxidative stress describes an imbalance in which reactive oxygen and nitrogen species (ROS/RNS) overwhelm the capacity of cellular antioxidant and repair systems. While low-level oxidant production is integral to physiological redox signaling, sustained excess ROS/RNS can oxidize lipids, proteins, and nucleic acids, disrupt organellar function, and reprogram gene expression through redox-sensitive transcriptional and epigenetic mechanisms. Exogenous stressors—including airborne particulate matter, ozone (O_3_), nitrogen dioxide (NO_2_), heavy metals, pesticides, and engineered nano-/micro-materials—can substantially amplify oxidant burden beyond that generated by endogenous sources such as mitochondrial electron transport, cytochrome P450 metabolism, and immune-cell nicotinamide adenine dinucleotide phosphate (NADPH) oxidases [1,2,3]. Epidemiological and experimental studies further suggest that chronic pollutant exposure contributes to cardiometabolic and neurodegenerative disease and may accelerate biological aging, particularly in older individuals whose antioxidant capacity and mitochondrial resilience are already diminished [4,5,6]. Despite the timeliness of this topic, the existing review literature is extensive. Prior reviews have often focused on (i) oxidative stress and epigenetic regulation, (ii) pollutant-driven epigenetic dysregulation, (iii) air pollution and biological aging, or (iv) oxidative-stress biomarkers in specific disease contexts. The added value of the present review is a pollutant-centered, cross-disciplinary synthesis that explicitly connects (a) pollutant-class–specific ROS-generation mechanisms, (b) the redox-sensitive epigenetic machinery that can “store” oxidative exposures as persistent changes in gene regulation, and (c) the practical problem of measuring these processes with biomarkers that are fit-for-purpose for human studies and, ultimately, clinical translation. Accordingly, this review pursues three goals. First, we compare mechanistic pathways across major pollutant classes to identify convergent ROS “nodes” (e.g., mitochondrial dysfunction, NADPH oxidase activation, and redox cycling/Fenton chemistry) and their downstream consequences. Second, we synthesize how oxidative stress interfaces with DNA methylation, histone modifications, and non-coding RNAs, with attention to where evidence is strongest (cell culture vs. animal vs. human observational studies) and where key controversies and gaps remain. Third, we critically evaluate oxidative-stress biomarkers and emerging redox-epigenetic and multi-omics signatures by analytical specificity, pre-analytical vulnerability, biological interpretability, and translational readiness, and we discuss how exposomics and AI-assisted integration may help—while also acknowledging current constraints that limit clinical deployment.

To increase reproducibility, we prioritized primary studies and high-quality consensus resources, emphasizing literature published from 2020 to 2025 while retaining seminal earlier work where needed for mechanistic grounding and biomarker validation.

## 2. Mechanisms of Oxidative Stress Induced by Environmental Pollutants

### 2.1. Airborne Pollutants

**Figure 1:**
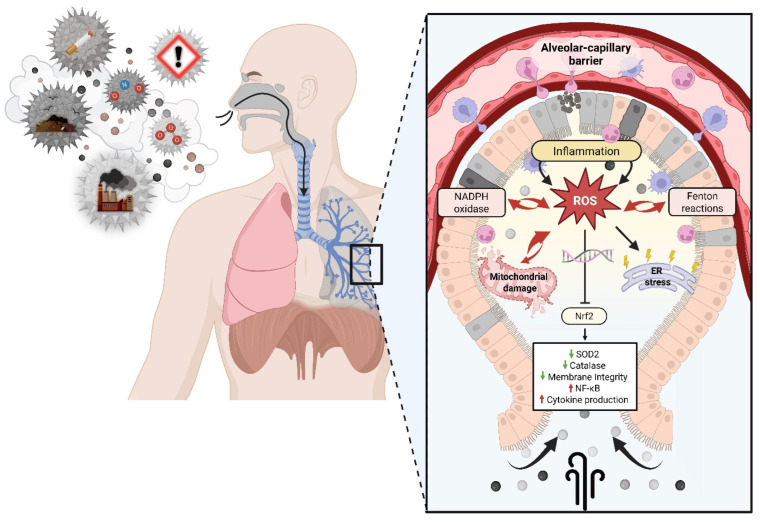
Schematic of airborne pollutant–induced oxidative stress. Airborne particulates and gaseous pollutants, such as nitrogen dioxide (NO_2_), are primarily emitted into the atmosphere by burning fossil fuels, leading to the production of harmful secondary air pollutants like ozone (O_3_). Exposure to decreased air quality allows pollutants to penetrate deep into the respiratory tract, causing Fenton reactions that generate ROS and stimulate NADPH oxidase activity within the alveolar-capillary barrier, where oxygen exchange occurs. Resultant oxidative stress disrupts mitochondrial ETC, promotes excessive mitochondrial fission, increases ER stress, and enhances neutrophil and macrophage transmigration. Chronic exposure leads to inflammation, lipid peroxidation, apoptosis, and systemic cardiovascular effects. Created in BioRender. González, M. (2026) https://BioRender.com/dj1v7is (accessed on 31 March 2026).

**Particulate matter and gases.** Particulate matter and gases. Fine particulate matter (PM_2.5_) and ultrafine particles (UFPs) deliver redox-active constituents (e.g., transition metals and redox-cycling organics) that can generate reactive species directly in airway lining fluid and after cellular uptake. Metals such as Fe and Cu catalyze Fenton chemistry (Fe^2+^ + H_2_O_2_ → Fe^3+^ + OH^−^ + ^•^OH), while quinones and related organics can undergo redox cycling, sustaining superoxide (O_2_^•−^) and hydrogen peroxide (H_2_O_2_) production [7,8]. Their small size also enables deep lung deposition and translocation across the alveolar barrier, where mitochondrial electron transport chain (ETC) disruption increases electron leakage and mitochondrial ROS production [9]. In parallel, gaseous oxidants such as ozone (O_3_) react with unsaturated lipids and antioxidants in the airway lining fluid to form secondary oxidation products (e.g., aldehydes and lipid ozonation products) that propagate oxidative injury [10]. A key conceptual distinction is between these proximal ROS-generating processes and the downstream biological amplification loops (innate immune activation, organellar stress, and redox-sensitive transcription) that sustain inflammation and tissue remodeling. In many experimental systems, low or intermittent oxidant exposures activate adaptive programs through the redox-sensitive transcription factor NRF2, whereas sustained exposure overwhelms antioxidant capacity and favors pro-inflammatory signaling (e.g., NF-κB activation), lipid peroxidation, and NADPH oxidase (NOX) up-regulation [11]. These major pathways are summarized in Figure 1.

**Traffic-related pollutants.** Chronic exposure to traffic-related pollutants sustains alveolar macrophage and neutrophil activation. These immune cells produce ROS via NADPH oxidase and myeloperoxidase as part of host defense, but in the context of environmental particles, the oxidative burst damages lung tissue and propagates inflammation [12]. Particulate matter often carries polycyclic aromatic hydrocarbons (PAHs), dioxins, and transition metals on its surface; after inhalation, these compounds can dissolve in airway fluids, partition into membranes, and contribute to secondary ROS formation [13,14]. Ozone (O_3_) -driven lipid ozonation products and NO_2_–associated nitrating chemistry can further increase oxidative and nitrosative stress; in inflamed tissue, superoxide can also react with nitric oxide (NO) to form peroxynitrite (ONOO^−^), a potent oxidant and nitrating species [10,15,16]. The resulting oxidant burden compromises epithelial barrier integrity, increases vascular permeability, activates coagulation pathways, and promotes systemic inflammation [17].

**Mitochondrial dysfunction and NOX activation.** Mitochondria are a primary source of endogenous ROS and a major target of pollutant-induced damage. Exposure to airborne particulate matter disrupts mitochondrial homeostasis by impairing the ETC, leading to electron leakage and superoxide overproduction [18]. Pollutants regulate mitochondrial dynamics by increasing the expression of dynamin-related protein 1 (DRP1) and decreasing mitofusin 1/2 (MFN1/2) and optic atrophy protein 1 (OPA1), resulting in excessive fission and mitochondrial fragmentation [19]. ROS accumulation triggers mitochondrial permeability transition pore (mPTP) opening, leading to cytochrome c (Cyt C) release and caspase-dependent apoptosis [20]. Oxidative damage to mitochondrial DNA (mtDNA) impairs replication and transcription of ETC subunits, exacerbating electron leakage [21]. Inhibition of peroxisome proliferator-activated receptor γ coactivator 1-α (PGC-1α) and nuclear respiratory factor 1 (NRF1) further impairs mitochondrial biogenesis [22], diminishing the cell’s capacity to recover from oxidative insults. Transition metals within PM_2.5_ catalyze Fenton and Haber–Weiss reactions, generating hydroxyl radicals that oxidize mitochondrial membranes and enzymes [14]. Chronic exposure leads to sustained mitochondrial ROS (mtROS) generation, establishing a self-amplifying oxidative loop that contributes to tissue injury and aging [23].

Beyond mitochondria, NADPH oxidases (NOX family enzymes) play a critical role in pollutant-induced ROS generation. PM_2.5_ and ozone exposure activate NOX1, NOX2, and NOX4 in epithelial cells, macrophages, and endothelial tissues via Toll-like receptor (TLR) and MAPK signaling [24,25,26]. This activation converts molecular oxygen into superoxide, which reacts with nitric oxide to form peroxynitrite, a highly reactive nitrating agent. NOX-derived ROS initiates oxidative modification of proteins, lipids, and DNA and activates transcription factors such as NF-κB and activator protein-1 (AP-1) [27]. These, in turn, up-regulate pro-inflammatory cytokines (IL-6, TNF-α, IL-1β), adhesion molecules (ICAM-1, VCAM-1), and chemokines that recruit immune cells to inflamed sites [24]. Chronic NOX activation contributes to vascular dysfunction, endothelial barrier disruption, and atherogenesis [28,29,30]. Pharmacological inhibition of NOX enzymes attenuates pollutant-induced oxidative damage and systemic inflammation [31,32,33].

Endoplasmic reticulum (ER) stress is an emerging hallmark of oxidative damage induced by airborne pollutants. ROS interfere with protein folding in the ER, triggering the unfolded protein response (UPR) [34]. Key molecular markers of UPR activation GRP78/BiP, C/EBP homologous protein (CHOP), and ATF4 are up-regulated following chronic PM_2.5_ exposure [35]. Persistent ER stress disrupts calcium homeostasis, leading to mitochondrial calcium overload and enhanced ROS generation via the mitochondria-associated membrane (MAM) interface [36]. This bidirectional signaling between mitochondria and the ER amplifies oxidative damage and promotes apoptosis. Pollutant exposure suppresses ER-associated degradation, resulting in protein aggregation and further oxidative injury [18]. The combination of mitochondrial dysfunction and ER stress drives chronic inflammation, fibrosis, and degenerative lung pathologies. In the lung, ER stress impairs surfactant secretion and promotes fibrotic remodeling [37]. Fine particles can translocate into circulation, uncouple endothelial nitric oxide synthase (eNOS), and reduce nitric oxide bioavailability, contributing to hypertension [38]. Oxidatively modified low-density lipoproteins (oxLDL) are generated in this milieu; they activate TLRs on macrophages, drive foam-cell formation, and accelerate atherosclerosis [39].

### 2.2. Heavy Metals and Pesticides

**Figure 2:**
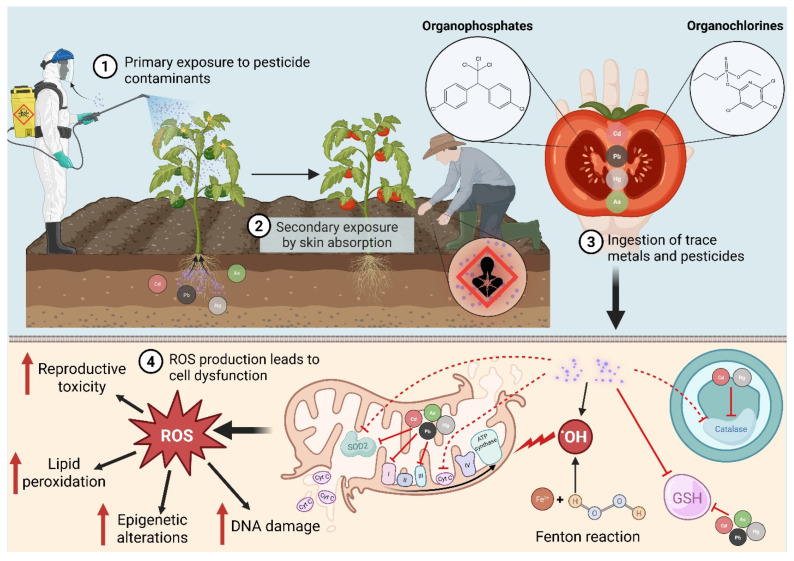
Pathways of heavy metal and pesticide-induced oxidative stress. Both primary and secondary exposure to pesticides disrupts mitochondrial respiration. The ingestion of redox and non-redox active trace metals generate hydroxyl radicals, deplete glutathione, and inhibit antioxidant enzymes. The resultant ROS triggers DNA damage, lipid peroxidation, epigenetic alterations (DNA methylation, histone modification, miRNA dysregulation), and reproductive toxicity. Created in BioRender. Powers, M. (2026) https://BioRender.com/84lc2vq (accessed on 31 March 2026).

**Redox cycling and ROS production.** Heavy metals such as iron and copper catalyze Fenton reactions that convert hydrogen peroxide into highly reactive hydroxyl radicals [40]. Non-redox-active metals like cadmium and mercury bind to sulfhydryl groups, deplete glutathione (GSH), and inactivate antioxidant enzymes such as superoxide dismutase (SOD) and catalase, thereby indirectly increasing ROS levels [41]. Chronic exposure to lead, arsenic, or cadmium interferes with mitochondrial complexes I and III, leading to electron leakage, superoxide accumulation, and suppression of mitochondrial biogenesis [42,43]. These convergent pathways are summarized in Figure 2.

Pesticides, particularly organophosphates and organochlorines, disrupt the ETC, collapse mitochondrial membrane potential, and increase cytochrome c release [44]. They also inhibit SOD, catalase, and glutathione peroxidase while increasing lipid peroxidation products such as malondialdehyde (MDA) and 4-hydroxynonenal (4-HNE). ROS generated by pesticides activate NF-κB, MAPK, and PI3K/Akt pathways, induce inducible nitric oxide synthase (iNOS), and promote DNA and protein nitration, amplifying inflammation and cell death [45].

Specific metal mechanisms. Cadmium displaces zinc from metalloproteins and binds to thiol groups in glutathione, reducing cellular GSH and increasing susceptibility to oxidative damage [46]. Lead inhibits δ-aminolaevulinic acid dehydratase, disrupting heme synthesis and causing anemia; it interferes with calcium signaling in neurons and compromises synaptic transmission [47]. Mercury forms strong complexes with selenoenzymes such as thioredoxin reductase, impairing the detoxification of hydrogen peroxide [48]. Arsenic metabolites inhibit pyruvate dehydrogenase and α-ketoglutarate dehydrogenase, leading to NADH accumulation, ATP depletion, and mitochondrial dysfunction [49].

Pesticide classes also differ in their redox effects. Organophosphates such as chlorpyrifos and malathion inhibit acetylcholinesterase, causing sustained neurotransmission and calcium overload; the resulting excitotoxicity increases mitochondrial ROS and triggers apoptosis [50]. Organochlorines such as DDT alter lipid metabolism and uncouple oxidative phosphorylation, increasing oxygen consumption and superoxide production [51]. Carbamates and triazine herbicides generate redox-active intermediates that oxidize proteins and DNA [52]. Many pesticides induce cytochrome P450 enzymes, bioactivating xenobiotics into electrophilic metabolites that form adducts with proteins and nucleic acids [53].

**Epigenetic and reproductive effects.** Heavy metals and pesticides modulate epigenetic machinery. Cadmium and arsenic alter DNA methylation patterns by inhibiting DNA methyltransferases (DNMTs) or depleting methyl donors, causing global hypomethylation and site-specific hypermethylation of tumor suppressor genes [54]. Pesticides modify histone acetylation and alter microRNA expression (e.g., miR-34a and miR-21), suppressing sirtuins and Nrf2 and aggravating oxidative stress [55]. Combined exposures often show synergistic toxicity: co-exposure to cadmium and neonicotinoid pesticides increases ROS, mitochondrial collapse, and DNA strand breaks beyond individual effects.

Reproductive tissues are particularly vulnerable to oxidative damage. In males, cadmium accumulates in testicular tissue, inhibits steroidogenic enzymes (3β- and 17β-hydroxysteroid dehydrogenases), reduces testosterone production, and damages Sertoli cells [56]. Lipid peroxidation of sperm plasma membranes decreases motility and alters DNA integrity, contributing to infertility [57]. Organophosphates suppress acetylcholinesterase in Leydig cells, leading to oxidative injury and reduced androgen synthesis [58]. In females, organochlorines accumulate in adipose tissue and ovarian follicles, disrupting estrogen signaling and ovulation [59]. Heavy metals cross the placenta, exposing the developing embryo to ROS and epigenetic alterations that may manifest as developmental defects or transgenerational effects [60].

Systemically, these toxicants contribute to neurotoxicity, endocrine disruption, insulin resistance, and hypertension via persistent redox imbalance and inflammation.

### 2.3. Nanoparticles and Microplastics

**Figure 3:**
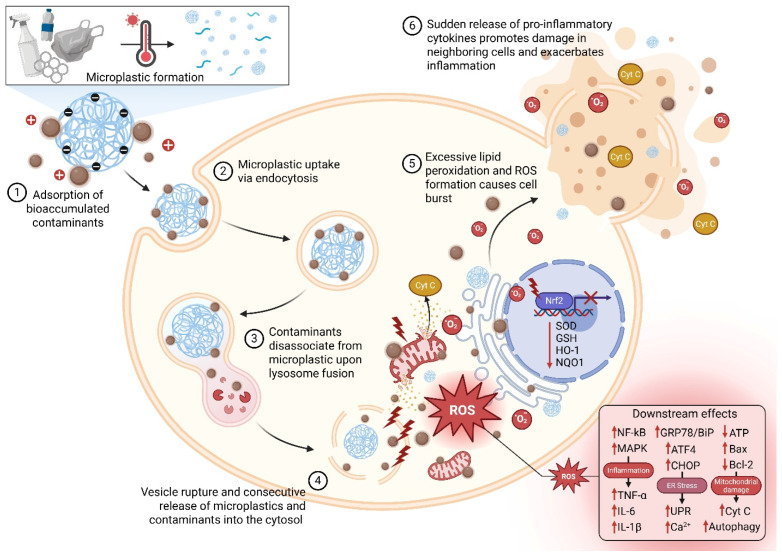
Mechanisms of nanoparticle and microplastic–induced oxidative stress. Plastic waste undergoes a lengthy process of environmental degradation and physical abrasion, creating microplastics and nanoparticles that can easily infiltrate into biological systems. Microplastics can act as vectors, adsorbing toxic chemicals and bioaccumulated contaminants via surface reactivity. Upon cell intake, microplastics along with the adsorbed contaminants, can begin generating ROS and cause damage to lipids, proteins, and DNA; disrupt mitochondria and ER; and activate inflammatory pathways. Created in BioRender. González, M. (2026) https://BioRender.com/6fimvww (accessed on 31 March 2026).

**ROS generation and mitochondrial injury.** Nanoparticles including TiO_2_, ZnO, Ag, and micro-/nanoplastics accumulate in organisms because of their small size and expansive surface area. Metal nanoparticles generate reactive oxygen species (ROS) via redox cycling, while non-metal particles contribute to ROS formation through surface-bound sites or contaminants [61]. As a result, ingested microplastics can disrupt cell membranes, increase ROS levels, cause mitochondrial dysfunction, and trigger inflammation, all of which weaken antioxidant defenses and lead to DNA damage in mitochondria and the nucleus [62,63]. Furthermore, metal nanoparticles release ions that disrupt organelles and induce oxidative stress. Materials like carbon nanotubes promote apoptosis through multiple cellular pathways including death receptor, ER stress, and mitochondrial functions [64]. Ultimately, chronic exposure to microplastics elevates ROS and oxidative stress, linking these processes to inflammation, respiratory diseases, cancer, and cellular aging. These processes are summarized in Figure 3.

**Micro- and nano-plastics as vectors.** Micro- and nano-plastics act as vectors for hydrophobic pollutants, heavy metals, and endocrine-disrupting chemicals. Their hydrophobic surfaces adsorb persistent organic pollutants (POPs) and heavy metals in the environment; after uptake by organisms, desorption of these contaminants in acidic intracellular compartments produces additional ROS and toxic effects [65]. Nano-sized plastic particles can cross biological barriers, including the blood–brain barrier, accumulating in brain regions such as the hippocampus where they trigger microglial activation, calcium imbalance, and neuronal death [66]. Size of these nanoparticle plays a major role as recent studies have shown that smaller polystyrene microplastics (<100 nm) cause more severe mitochondrial swelling, ATP depletion, and DNA strand breaks than larger ones, underscoring the importance of particle size [67]. Ultimately, chronic exposure to microplastics raises ROS and oxidative stress, contributing to inflammation, pulmonary disease, cancer, and aging [62].

**Inflammatory signaling and systemic effects.** ROS generated by nanoparticles and microplastics activate NF-κB and MAPK cascades, leading to cytokine release and chronic inflammation [68]. At first, Nrf2 activation induces antioxidant enzymes (HO-1, NQO1), but prolonged exposure exhausts this response, leaving cells susceptible to oxidative damage [11]. Nanoparticles rapidly adsorb proteins, forming a “protein corona” that dictates cellular uptake and stimulates macrophage activation, producing an oxidative burst [69]. Microplastics disrupt gut microbiota, compromise intestinal barrier integrity, and accumulate in the liver and brain, where they induce lipid peroxidation and mitochondrial injury [70]. Co-exposure to microplastics and contaminants such as per- and polyfluoroalkyl substances (PFAS) or heavy metals enhances ROS generation and inhibits antioxidant defenses, resulting in synergistic toxicity [71,72].

ER stress and autophagy are intimately linked to nanoparticles and microplastic toxicity. Exposure to these particles upregulates ER stress markers (GRP78/BiP, ATF4, CHOP) and activates the UPR [73]. Persistent UPR causes calcium dysregulation and promotes crosstalk with mitochondria, increasing ROS and triggering apoptosis. Autophagy serves as an adaptive response to clear damaged mitochondria and proteins; however, chronic exposure impairs autophagic flux, leading to the accumulation of autophagosomes and cell death [74]. Dysbiosis of the gut microbiome caused by microplastics reduces commensal bacteria that produce antioxidant metabolites and increases pathogenic species that generate ROS [75]. Adaptive antioxidant responses, such as increased GSH synthesis and Nrf2 activation, initially help protect against stress; however, with prolonged exposure, these defenses become depleted. To address this, potential therapies include dietary antioxidants, melatonin, and engineered nanoparticles like cerium oxide. Additionally, nanocarriers delivering Nrf2 activators or mitochondrial-targeted antioxidants (such as MitoQ and SkQ1) have shown promise in combatting the oxidative damage induced by nanoparticles and microplastics [76].

### 2.4. Cross-Cutting Convergence and Open Questions

Although pollutant classes differ in chemistry, exposure route, and tissue distribution, several mechanistic themes recur across the literature reviewed here. First, diverse exposures converge on mitochondrial injury (ETC disruption, altered fission/fusion balance, mtDNA damage) that sustains mitochondrial ROS. Second, pattern-recognition and stress signaling pathways can activate NADPH oxidases, amplifying oxidant generation downstream of the initiating exposure. Third, organellar stress responses (ER stress, impaired autophagy/mitophagy) and inflammatory feedback loops can maintain oxidative stress even after the proximal exposure has ceased. A key unresolved question across these domains is the extent to which observed epigenetic alterations represent causal mediators of aging phenotypes versus correlated marks of exposure/inflammation; resolving this requires more longitudinal human data with standardized exposure assessment and fit-for-purpose biomarker measurements.

## 3. Epigenetic Modifications Driven by Oxidative Stress

**Figure 4:**
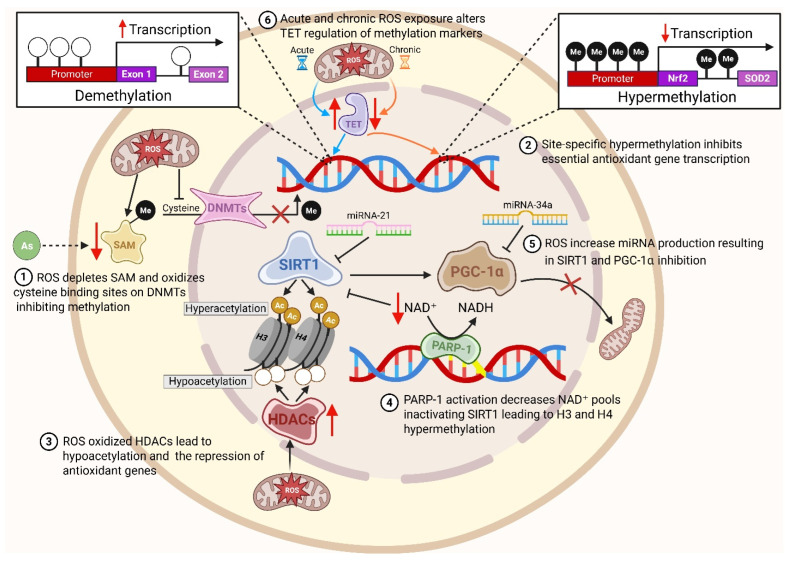
Epigenetic alterations triggered by oxidative stress. ROS oxidize DNMTs and deplete S-adenosyl-methionine (SAM), resulting in global hypomethylation while site-specific hypermethylation silences antioxidant genes. ROS-induced PARP-1 activation suppresses SIRT1, leading to histone hyperacetylation. Oxidative inhibition of histone deacetylases increases the repression of antioxidant genes via histone-wide hypoacetylation. Non-coding RNAs such as miR-21 and miR-34a suppress sirtuins and PGC-1α, integrating redox signals with epigenetic regulation. Acute and chronic ROS exposure leads to DNA demethylation and hypermethylation via TET regulation, respectively. Created in BioRender. Powers, M. (2026) https://BioRender.com/luzw8u5 (accessed on 31 March 2026).

Epigenetic mechanisms, including DNA methylation and hydroxymethylation, histone modifications, and non-coding RNA regulation, translate oxidative signals into stable changes in gene expression. The following sections retain detailed descriptions of redox-epigenetic cross-talk. Key redox–epigenetic interfaces are summarized in Figure 4.

### 3.1. DNA Methylation and Hydroxymethylation

DNA methylation is mediated by DNMT1, DNMT3A, and DNMT3B, which transfer methyl groups from S-adenosyl-methionine (SAM) to cytosine residues at CpG sites. Oxidative stress oxidizes catalytic cysteines in DNMTs and depletes the methyl donor SAM, leading to global hypomethylation and impaired maintenance methylation [77]. Oxidized guanine lesions (8-oxo-dG) inhibit DNMT binding, further reducing methylation density [78]. Conversely, transient ROS exposure increases ten-eleven translocation (TET) enzyme activity and 5-hydroxymethylcytosine levels, promoting active demethylation [79]. Chronic oxidative environments inhibit TET enzymes by depleting cofactors (Fe^2+^ and α-ketoglutarate), causing site-specific hypermethylation of tumor suppressor genes and impaired antioxidant gene expression [80]. Aging and chronic exposure to pollutants accelerate “epigenetic drift” stochastic changes in methylation that accumulate over time which silences antioxidant genes such as Nrf2 and SOD2 and perpetuates oxidative injury [81].

Environmental toxicants often cause promoter hypermethylation of genes involved in redox homeostasis; exposure to particulate matter, lead, or diesel exhaust is associated with increased methylation of the Nrf2 promoter and decreased expression of its downstream antioxidant genes. Mutations in isocitrate dehydrogenase (IDH) produce the oncometabolite 2-hydroxyglutarate, which inhibits TET enzymes and promotes widespread DNA hypermethylation and ROS generation [82]. Pollutants that alter one-carbon metabolism (e.g., arsenic) deplete folate and methionine pools, reducing the availability of SAM and further perturbing DNA methylation [54]. These redox-dependent methylation changes are reversible, suggesting therapeutic potential for methyl donor supplementation (folate, betaine) or DNMT inhibitors [83].

DNA methylation changes also serve as environmental biomarkers. Chronic exposure to cadmium, lead, and PM_2.5_ disrupts methylation of antioxidant and stress-response genes (Nrf2, HO-1, SOD2) [84]. Traffic-related air pollution induces hypomethylation of mitochondrial genes and hypermethylation of DNA repair genes, as reported in the Hortega cohort study [85]. These methylation changes correlate with increased 8-hydroxy-2′-deoxyguanosine (8-OHdG) levels, indicating oxidative DNA damage. Hypomethylation of Nrf2 and SOD2 and hypermethylation of DNA repair genes thus represent sensitive, reversible indicators of environmental oxidative load.

### 3.2. Histone Modifications

Histone acetylation is a key mechanism by which chromatin accessibility and gene expression are regulated, orchestrated by the opposing activities of histone acetyltransferases (HATs) and histone deacetylases (HDACs). Under conditions of oxidative stress, the function of these histone-modifying enzymes can be profoundly altered. For instance, oxidative activation of PARP-1 during DNA repair leads to NAD^+^ depletion, which in turn suppresses the activity of SIRT1, a NAD^+^-dependent deacetylase. This suppression results in hyperacetylation of histones H3 and H4 and disrupts normal transcriptional control [86,87]. Meanwhile, sustained exposure to reactive oxygen species (ROS) can activate class I and II HDACs, causing histone hypoacetylation and silencing antioxidant gene expression [88]. ROS also impact histone methylation by inhibiting demethylases such as KDM6B, thereby increasing repressive methylation marks like H3K27me3 and H3K9me3, which further silence stress-response genes [89]. Additionally, peroxynitrite-mediated nitration of histone residues can activate pro-inflammatory gene expression, highlighting the complex, dual regulatory roles of redox modifications in chromatin dynamics [90].

Beyond acetylation and methylation, oxidative stress influences a variety of other histone acylation modifications. Metabolic byproducts such as succinate and fumarate, which accumulate during mitochondrial dysfunction, can inhibit α-ketoglutarate-dependent histone demethylases and promote alternative acylations like histone succinylation and crotonylation [91]. These post-translational modifications serve as a direct link between cellular metabolic status and chromatin structure, either repressing or activating gene transcription depending on the context. ROS can also oxidize histone cysteine residues, disrupt disulfide bonds, and destabilize nucleosomes, further affecting chromatin integrity. Environmental exposures have been shown to enhance repressive H3K27me3 marks at promoters of detoxifying enzymes (such as HO-1 and NQO1) and reduce acetylation at enhancers of antioxidant genes [92]. Therapeutic strategies are being developed to counteract these changes, including pharmacological HDAC inhibitors (like trichostatin A and valproic acid) and sirtuin activators (such as resveratrol), which aim to restore histone acetylation and boost antioxidant gene expression [93,94,95].

### 3.3. Non-Coding RNAs

Non-coding RNAs play a pivotal role in connecting redox signaling to gene regulation. Under conditions of oxidative stress, microRNAs such as miR-21, miR-34a, and miR-200c are upregulated, which in turn suppress key regulators like SIRT1, PGC-1α, and Nrf2. This suppression hampers mitochondrial biogenesis and diminishes antioxidant defenses, making cells more vulnerable to oxidative damage [96]. Conversely, the down-regulation of microRNAs such as miR-25 and miR-146a lifts the inhibition on pro-inflammatory pathways, thereby intensifying inflammatory responses [97]. Long non-coding RNAs (lncRNAs), including MALAT1, HOTAIR, and ANRIL, are also sensitive to changes in redox status. For example, MALAT1 can facilitate the nuclear translocation of Nrf2 under moderate oxidative conditions, enhancing antioxidant responses. In contrast, persistent oxidative stress stimulates HOTAIR, which recruits chromatin repressors and silences tumor suppressor genes, contributing to disease progression [98,99]. Additionally, redox-sensitive circular RNAs (circRNAs) can act as microRNA sponges or interact directly with transcriptional machinery, thereby modulating oxidative signaling pathways [100].

These interconnected networks of non-coding RNAs orchestrate both adaptive and maladaptive cellular responses to oxidative stress and are increasingly recognized as promising therapeutic targets. Recent studies reveal that circRNAs often carry N6-methyladenosine (m^6^A) modifications, which are regulated by the cellular redox environment and significantly impact circRNA stability and translation [101]. Modulating ncRNA pathways using antisense oligonucleotides or small-molecule drugs holds great potential for restoring redox balance and maintaining epigenetic integrity, offering novel strategies for disease intervention.

### 3.4. Epigenetic Modifications in Disease Contexts

Oxidative stress plays a pivotal role in the pathogenesis of various diseases by influencing epigenetic modifications. In autoimmune disorders, elevated ROS levels drive T-cell demethylation of genes such as CD70 and ITGAL, which heightens autoreactive responses and fuels chronic inflammation [102]. Furthermore, ROS-mediated oxidation impairs the activity of TET enzymes, disrupting global 5-hydroxymethylcytosine patterns. This contributes to abnormal gene activation observed in conditions like systemic lupus erythematosus and rheumatoid arthritis [103]. Within cancerous environments, persistent oxidative stress leads to hypermethylation of tumor suppressor genes—including BRCA1 and p16^INK4a^—while simultaneously causing hypomethylation of oncogenes. These changes promote genomic instability and facilitate tumor progression [104]. Additionally, ROS stimulate DNMT3A and EZH2, resulting in chromatin condensation and transcriptional silencing of apoptotic genes, which further supports cancer cell survival [105]. In metabolic syndrome, diabetes, and atherosclerosis, oxidative stress induces hypermethylation of mitochondrial genes and deacetylation of key metabolic regulators, collectively impairing energy metabolism and endothelial function [106].

Neurodegenerative diseases and aging are also profoundly affected by redox-driven epigenetic changes. ROS exposure suppresses the expression of critical genes like BDNF and SOD2 through promoter hypermethylation, contributing to neuronal degeneration and loss [107]. Chronic oxidative stress alters histone acetylation marks, specifically H3K9 and H4K16, which impairs synaptic plasticity and disrupts long-term memory formation [108]. Collectively, these examples highlight the integral role of redox-epigenetic cross-talk in human disease. The identification of epigenetic alterations as both diagnostic biomarkers and therapeutic targets offers promising avenues for intervention in oxidative stress-related pathologies.

## 4. Oxidative Stress and Aging

**Figure 5:**
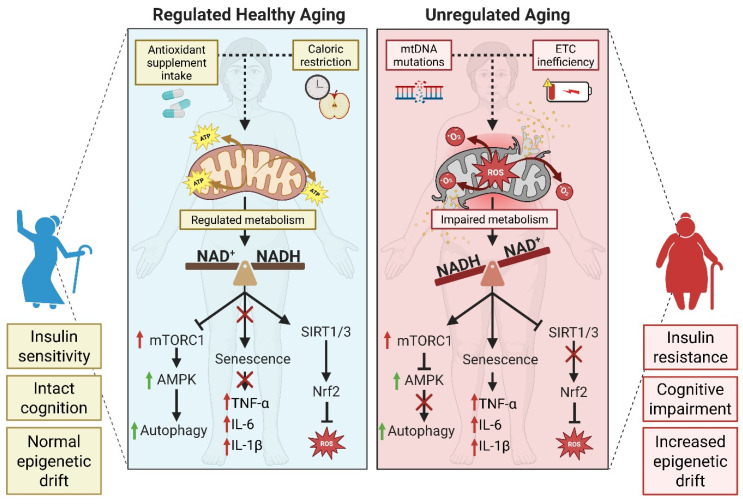
Interplay between oxidative stress and aging. The accumulation of mtDNA mutations and increased electron leakage due to ETC inefficiency in aging mitochondria boost ROS production over time. The consequential long-term impaired metabolism can result in a decline in NAD^+^/NADH ratios, inducing mTORC1 activation, SIRT1/3 inactivation, cellular senescence, and chronic inflammation. Unregulated aging can be accompanied by insulin resistance, cognitive impairment, and increased epigenetic drift. Healthy aging can be achieved by regulating ROS production with the activation of sirtuin pathways and Nrf2 transcription with the regulated intake of antioxidant supplements (NAD^+^ precursors), exercise, and caloric restriction. Created in BioRender. González, M. (2026) https://BioRender.com/lszhxip (accessed on 31 March 2026).

Aging is marked by a gradual decline in physiological resilience, closely tied to increased production of reactive oxygen species (ROS), mitochondrial dysfunction, and persistent inflammation. Mitochondria play a central role as both the main producers and primary targets of ROS. Under normal conditions, the electron transport chain (ETC) generates modest levels of ROS, which are efficiently neutralized by antioxidant enzymes such as SOD2 and glutathione peroxidase (GPx). However, as individuals age, inefficiencies in the ETC and mutations in mitochondrial DNA (mtDNA) lead to greater electron leakage, resulting in excessive superoxide formation and progressive mitochondrial damage [109]. Damaged mitochondria activate inflammatory signaling pathways and trigger the senescence-associated secretory phenotype (SASP), amplifying systemic inflammation—a phenomenon known as “inflammaging” [110]. Elevated oxidative stress further harms nuclear and mitochondrial DNA, proteins, and lipids, driving cellular senescence and shortening telomeres. Age-related reductions in NAD^+^ levels compromise sirtuin function and DNA repair mechanisms, while redox imbalances alter the activity of epigenetic enzymes, resulting in epigenetic drift and disrupted gene regulation [110]. Strategies that aim to restore mitochondrial function, maintain NAD^+^ concentrations, and activate Nrf2 and sirtuin pathways are increasingly recognized as crucial for promoting healthy aging and extending the health span [111]. These interconnected mechanisms are summarized in Figure 5.

The accumulation of ROS initiates DNA damage responses that stabilize the tumor suppressor protein p53 and activate cyclin-dependent kinase inhibitors, including p21^Cip1^ and p16^INK4a^. This activity enforces cell cycle arrest and supports the development of senescence [112]. Senescent cells secrete a diverse mix of pro-inflammatory cytokines, matrix metalloproteinases, and growth factors—collectively known as the SASP—which perpetuate inflammation and contribute to tissue degeneration [113]. Oxidative stress also influences nutrient-sensing pathways, such as mTOR, while inhibiting AMP-activated protein kinase (AMPK), thereby impacting autophagy and energy homeostasis [114]. In blood vessels, ROS decrease endothelial nitric oxide synthase (eNOS) activity, reduce nitric oxide availability, and promote vascular stiffness and hypertension. Meanwhile, in the brain, oxidative damage accelerates amyloid-β aggregation and tau phosphorylation, driving Alzheimer’s disease progression. In dopaminergic neurons, oxidative metabolism of dopamine produces quinones that contribute to the pathogenesis of Parkinson’s disease [115].

Metabolic tissues are similarly affected by oxidative stress. In skeletal muscle and adipose tissue, ROS disrupt insulin receptor signaling and GLUT4 translocation, leading to insulin resistance [116]. Lipid peroxidation products, such as 4-hydroxynonenal (4-HNE), form adducts with insulin signaling proteins, further impairing their function [117]. In the liver, ROS promote fat accumulation (steatosis) by activating SREBP-1c and inhibiting fatty acid oxidation [118]. Age-dependent declines in NAD^+^ reduce the activity of sirtuins, particularly SIRT1 and SIRT3, compromising mitochondrial function and the deacetylation of key regulators like PGC-1α, FOXO3a, and p53. Supplementing with NAD^+^ precursors—including nicotinamide riboside and nicotinamide mononucleotide—restores sirtuin activity, enhances mitochondrial health, and has been shown to extend lifespan in animal studies [119].

Excessive ROS stabilize NRF2, leading to increased expression of antioxidant enzymes and supporting the free radical theory of aging, which posits that aging is associated with elevated ROS and diminished mitochondrial enzyme activity [120]. Young mitochondria produce ROS only when stimulated, while aged mitochondria generate high ROS levels continuously. Communication between mitochondrial ROS and AMPK triggers antioxidant defenses [109,121]. Mild activation of the mitochondrial unfolded protein response is beneficial and extends lifespan, whereas excessive activation accelerates cellular aging. The SIRT2–p66Shc–mtROS pathway regulates vascular aging, and AMPK activation reduces mitochondrial ROS. These findings underscore the importance of tightly regulated ROS signaling for cellular homeostasis, with chronic oxidative stress accelerating the aging process [122].

Therapeutic approaches to counteract oxidative aging include activating Nrf2 with phytochemicals like sulforaphane and curcumin, supplementing antioxidants such as vitamin E and coenzyme Q10, and employing mitochondrial uncouplers to reduce ROS production. Senolytic drugs selectively eliminate senescent cells, while mesenchymal stem cell–derived exosomes, which carry antioxidant enzymes and microRNAs, enhance autophagy and decrease ROS in aged tissues. Lifestyle interventions like caloric restriction and intermittent fasting lower oxidative damage by reducing mitochondrial ROS output and activating AMPK. Regular exercise strengthens endogenous antioxidant systems, stimulates mitochondrial biogenesis, and improves insulin sensitivity. Emerging therapies seek to combine sirtuin activators, NAD^+^ precursors, and Nrf2 inducers to restore redox balance and promote longevity [123,124].

## 5. Biomarkers and Measurement Tools

Quantifying oxidative stress is essential for risk assessment and therapeutic monitoring; however, no single biomarker captures the full complexity of redox imbalance. We therefore emphasize panels that integrate complementary endpoints across lipid, protein, DNA, and metabolic redox domains [125]. A central challenge for interpretation is that commonly used assays differ markedly in analytical specificity, susceptibility to artefactual oxidation, and biological interpretability. To increase translational clarity, we distinguish (i) relatively well-validated oxidative damage markers that can be quantified with high analytical specificity in human samples (e.g., mass-spectrometry–based F_2_-isoprostanes and 8-OHdG), (ii) research-grade or context-dependent markers (e.g., TBARS-derived MDA estimates, fluorescent ROS probes), and (iii) functional redox readouts (e.g., GSH/GSSG and NAD^+^/NADH ratios) that require strict pre-analytical control (see also Table 1). Below, we summarize key biomarkers alongside the analytical methods most commonly used to quantify them and highlight practical constraints that affect cross-study comparability.

### 5.1. Lipid Peroxidation Markers

**Malondialdehyde (MDA).** MDA is a three-carbon dialdehyde produced during the peroxidation of polyunsaturated fatty acids. It is one of the most frequently reported lipid peroxidation markers in clinical studies [126]. MDA is typically measured using the thiobarbituric acid reactive substances (TBARS) assay, in which MDA condenses with thiobarbituric acid at high temperature and acidic pH to form a colored adduct that is quantified spectrophotometrically. Although the TBARS assay is rapid and inexpensive, it lacks specificity because thiobarbituric acid also reacts with other aldehydes and lipid oxidation products, and MDA can be generated during the assay itself. High-performance liquid chromatography (HPLC) or gas chromatography coupled with mass spectrometry (GC-MS) provides greater specificity by separating MDA–TBA adducts from interfering substances [127,128]. Due to these limitations, TBARS values are often reported as total TBA-reactive species rather than absolute MDA concentrations. Elevated MDA/TBARS levels are reported in cardiovascular diseases, neurodegeneration, psychiatric disorders, and chronic kidney disease; however, variability across studies underscores the need for careful sample handling and analytical standardization [129].

**F_2_-isoprostanes (IsoPs).** These prostaglandin-like compounds are formed via free radical–mediated peroxidation of arachidonic acid. Because IsoPs are generated in situ from phospholipid-bound fatty acids and released by phospholipases, their levels accurately reflect endogenous lipid peroxidation independent of cyclooxygenase activity [130]. Multiple studies, including the NIH-sponsored Biomarkers of Oxidative Stress Study, have shown that quantification of F_2_-IsoPs provides one of the most reliable indices of oxidative stress in vivo [131]. IsoPs are remarkably stable and detectable in plasma, urine, cerebrospinal fluid, and exhaled breath condensate. Their measurement typically involves solid-phase extraction, purification by thin-layer chromatography, and quantification by GC-MS or LC-MS, often using negative ion chemical ionization and stable isotope dilution to achieve picogram-level sensitivity [132]. Alternatively, enzyme immunoassays are available but may lack specificity for individual IsoP isomers. Elevated IsoPs are observed in conditions ranging from atherosclerosis and diabetes to neurodegenerative and inflammatory diseases, and decrease in response to antioxidant therapy [133].

**4-Hydroxynonenal (4-HNE).** This highly reactive α,β-unsaturated aldehyde forms Michael adducts with proteins and phospholipids. 4-HNE levels are assessed using ELISA, HPLC, or Western blotting of 4-HNE–protein adducts. Due to its cytotoxicity, 4-HNE is both a biomarker and mediator of oxidative damage. Elevated levels are seen in neurodegenerative disorders, liver disease, diabetes, and atherosclerosis [134].

### 5.2. DNA Oxidation Products

**8-Hydroxy-2′-deoxyguanosine (8-OHdG) and 8-hydroxyguanine (8-OHGua).** These biomarkers arise when ROS oxidize guanine in DNA or the nucleotide pool. Urinary 8-OHdG is one of the most popular biomarkers of oxidative DNA damage. In vivo, oxidized guanine is excised by DNA glycosylases and excreted in urine; thus, urinary levels reflect whole-body DNA repair [135]. 8-OHdG/8-OHGua can also be measured in plasma, saliva, or tissue samples. Analytical techniques include competitive ELISA, which is simple but susceptible to cross-reactivity and tends to overestimate concentrations, and chromatographic methods such as HPLC coupled with electrochemical detection or tandem mass spectrometry, which provide higher specificity and sensitivity [136]. A column-switching HPLC-ECD system has been used to quantify 8-OHGua in saliva; salivary 8-OHGua was detectable at picogram levels and increased in smokers compared with non-smokers [137]. Regardless of matrix, rigorous sample preparation and avoidance of artefactual oxidation are crucial for reliable measurement [138].

### 5.3. Protein Oxidation Markers

**Protein carbonyls.** Oxidative modification of protein side chains yields carbonyl groups that accumulate with age and disease. Derivatization with 2,4-dinitrophenylhydrazine (DNPH) allows spectrophotometric quantification of carbonyls. Carbonylated proteins can also be separated by HPLC or detected by ELISA using anti-DNP antibodies. Western blotting following DNPH derivatization enables identification of specific oxidized proteins, while mass spectrometry provides precise structural information [139]. Plasma protein carbonyls increase in chronic kidney disease, diabetes, and neurodegenerative disorders, although levels vary depending on sample handling and analytical method [140].

**3-Nitrotyrosine and dityrosine.** Nitration and cross-linking of tyrosine residues by reactive nitrogen species produce 3-nitrotyrosine (3-NO_2_-Tyr) and dityrosine (diTyr). High-performance liquid chromatography coupled with electrochemical array detection (HPLC-ECD) enables simultaneous quantification of tyrosine, 3-NO_2_-Tyr, and diTyr in tissues and fluids. An Alzheimer’s disease study showed that dityrosine and 3-NO_2_-Tyr were elevated five- to eight-fold in hippocampal and neocortical regions compared with controls, whereas uric acid, a peroxynitrite scavenger, was decreased [141]. These findings illustrate the value of nitrosative stress markers for linking inflammatory enzyme activity to disease pathology. Immunochemical methods (ELISA, immunohistochemistry) are also used but may lack quantitative accuracy [142].

**Advanced oxidation protein products (AOPPs).** These dityrosine-containing cross-linked protein aggregates are formed by the action of chlorinated oxidants and myeloperoxidase-derived species. AOPPs are measured spectrophotometrically at 340 nm and correlate with systemic inflammation, especially in chronic renal failure and cardiovascular disease. Beyond serving as markers, AOPPs act as pro-inflammatory mediators that activate mononuclear phagocytes. Because protein oxidation products are more stable than lipid peroxidation products, AOPPs provide a robust index of chronic oxidative injury. Tissue or plasma AOPP concentrations are commonly measured by a spectrophotometric method in which oxidation of potassium iodide by chloramine-T yields triiodide ions; absorbance at 340 nm is compared against chloramine-T standards and expressed as nmol of chloramine-T equivalents per mg protein. Commercial ELISA kits are available for high-throughput screening [143].

**Ischemia-modified albumin (IMA).** Oxidative cleavage and conformational changes at the N-terminus of human serum albumin under ischemic or oxidative stress conditions reduce metal-binding capacity. The albumin cobalt-binding (ACB) test indirectly quantifies IMA by adding a known amount of cobalt and measuring the unbound fraction using a chromogenic agent such as dithiothreitol. Elevated IMA levels have been reported in acute coronary syndromes, sepsis, liver cirrhosis, and dermatological disorders. Limitations include reagent instability and interference by fatty acids or albumin variants; alternative colorimetric assays using nickel ions have been proposed to improve robustness [144,145,146].

### 5.4. Antioxidant Enzymes and Redox Cofactors

**Antioxidant enzymes.** Activities of endogenous antioxidant enzymes provide functional information on the oxidative state. SOD catalyzes the dismutation of superoxide to hydrogen peroxide; catalase and glutathione peroxidase further detoxify hydrogen peroxide to water. In tissues, SOD activity can be measured spectrophotometrically by assessing inhibition of nitroblue tetrazolium reduction in the xanthine–xanthine oxidase system. Catalase activity is often determined by monitoring the decomposition rate of hydrogen peroxide at 240 nm, while glutathione peroxidase activity is measured by coupled assays that monitor NADPH consumption during reduction of oxidized glutathione. Decreased enzymatic activity reflects depletion or inactivation of antioxidants and is frequently reported alongside increased oxidative stress biomarkers [147,148].

**Direct ROS detection.** Because free radicals have very short half-lives, direct measurement relies on trapping or fluorescent probes. 2′,7′-Dichlorodihydrofluorescein diacetate (DCFH-DA) is a cell-permeable probe that is deacetylated by intracellular esterases and oxidized by ROS to form fluorescent dichlorofluorescein. A standard DCFH-DA assay involves incubating cells with a 10 µM solution, washing, and measuring fluorescence at 485 nm excitation and 530 nm emission. This method provides a simple, cost-effective measure of total ROS generation but is not specific to individual ROS and can be influenced by light exposure and cell type. Electron spin resonance (ESR) spectroscopy combined with spin-trapping agents (e.g., DMPO) offers a more selective approach by forming stable radical adducts that can be detected directly, although it requires specialized instrumentation [149,150].

**Glutathione (GSH/GSSG) ratio.** GSH is the primary cellular thiol antioxidant, and its oxidation to glutathione disulfide (GSSG) reflects oxidative pressure. Under oxidative stress, GSH is consumed, and the GSH:GSSG ratio decreases; this shift serves as a sensitive indicator of redox status across diseases. Measurement can be performed by enzymatic recycling assays using glutathione reductase, HPLC with fluorescence detection after derivatization with orthophthalaldehyde, or capillary electrophoresis. Because the pool of protein-bound glutathione (S-glutathionylation) also increases under oxidative stress, assays that differentiate free and protein-bound glutathione provide more comprehensive information [151].

**NAD^+^/NADH ratio.** This redox pair underpins metabolic flux through glycolysis, the tricarboxylic acid (TCA) cycle, and oxidative phosphorylation. NAD^+^ accepts electrons and becomes reduced to NADH, which in turn donates electrons to the electron transport chain. A decline in the NAD^+^/NADH ratio is indicative of impaired mitochondrial respiration and is a hallmark of oxidative stress and aging. This ratio also regulates the activity of sirtuins (NAD^+^-dependent deacetylases), PARPs, and other redox-sensitive enzymes. The NAD^+^/NADH ratio is measured in tissues and cells using enzymatic cycling assays or LC-MS/MS. Pre-analytical variables, including sample storage, pH, and redox-active metabolites, must be strictly controlled for reliable quantification. NAD^+^ precursors such as nicotinamide riboside (NR) and nicotinamide mononucleotide (NMN) are currently being explored in clinical trials to restore NAD^+^ pools and mitigate oxidative damage [152,153].

## 6. Emerging Epigenetic Biomarkers and Redox-Related Metabolomic Profiles

**Figure 6:**
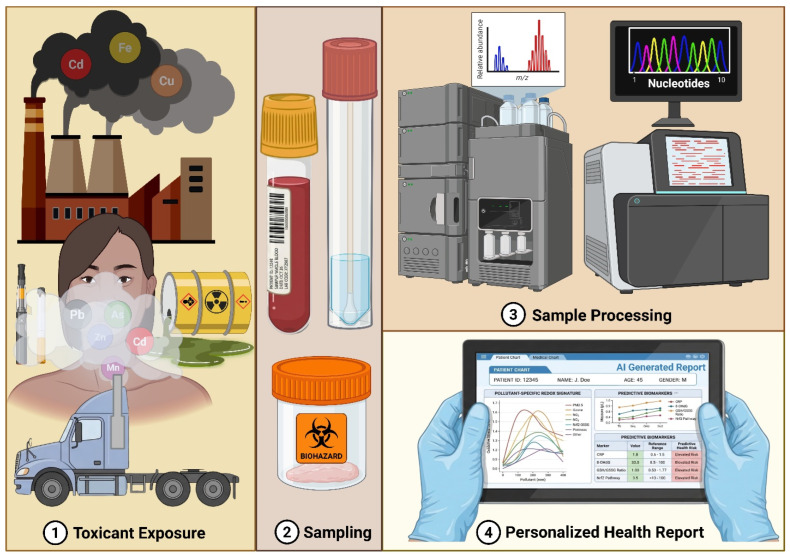
Conceptual multi-omics workflow for discovery of redox-epigenetic signatures of environmental exposures. Environmental toxicant exposures can generate oxidative stress, altering metabolites, DNA methylation, histone modifications, and non-coding RNA expression. Multi-omics integration can be used to nominate pollutant-associated candidate signatures and generate mechanistic hypotheses; however, translation to clinical decision-making requires explicit context-of-use definition, standardized biospecimen handling, robust analytical pipelines, and independent prospective validation. Created in BioRender. Powers, M. (2026) https://BioRender.com/cjqislh (accessed on 31 March 2026).

Recent advances in epigenomics and metabolomics have clarified how oxidative stress can modify gene regulation and metabolism, producing measurable molecular signatures that may serve as exposure or effect biomarkers. This section emphasizes the biomarker perspective: which redox-epigenetic and metabolomic features are detectable in accessible human biospecimens, what they plausibly represent mechanistically, and what key limitations currently constrain interpretation and clinical translation. Importantly, most epigenetic and multi-omics signatures discussed below remain exploratory; their value often lies in hypothesis generation, exposure stratification, or mechanistic anchoring of established oxidative-damage markers (Section 5), rather than near-term clinical decision-making. An overview of representative marker classes and use cases is provided in Table 2, and the conceptual multi-omics discovery workflow is illustrated in Figure 6.

### 6.1. DNA Methylation as a Redox Biomarker

DNA methylation changes are sensitive markers of environmental oxidative load. Exposure to cadmium, lead, and particulate matter disrupts methylation of antioxidant and stress-response genes (*Nrf2*, *HO-1*, *SOD2*) [154]. These methylation changes correlate with increased 8-OHdG levels, indicating oxidative DNA damage. Traffic-related air pollution induces hypomethylation of mitochondrial genes and hypermethylation of DNA repair genes, as reported in the Hortega cohort study, which linked environmental exposure to altered metabolite profiles and oxidative stress biomarkers [85]. Hypomethylation of *Nrf2* and *SOD2* and hypermethylation of DNA repair genes thus represent sensitive, reversible indicators of environmental oxidative load.

### 6.2. Histone Modifications and Chromatin Remodeling

Histone modifications respond dynamically to ROS. Heavy metals or PAHs induce repressive marks (H3K27me3, H3K9me3) that suppress expression of detoxifying genes [91,155]. Histone succinylation and crotonylation, new oxidative-sensitive modifications, link metabolic status to chromatin architecture. Environmental stress disrupts the acetyl-CoA and succinyl-CoA pools, altering histone acylation states and transcriptional control of antioxidant pathways. Non-redundant modifications such as histone succinylation and crotonylation are gaining attention for their ability to link metabolism to epigenetic control [156]. Emerging evidence highlights how metabolic intermediates such as fumarate and succinate modulate TET and KDM enzyme activity, establishing a direct biochemical link between metabolism and chromatin regulation [91].

### 6.3. Non-Coding RNA Regulation Under Redox Stress

Non-coding RNAs, especially microRNAs (miRNAs), long non-coding RNAs (lncRNAs), and circular RNAs (circRNAs), act as epigenetic regulators in oxidative stress responses [157]. ROS modulate miRNAs that target antioxidant and mitochondrial genes. miR-34a, miR-21, and miR-155 are consistently up-regulated by ROS and regulate sirtuins, Nrf2, and inflammatory pathways [158,159,160]. Circulating miRNA profiles reflect redox balance and offer non-invasive biomarkers for environmental exposure [161]. LncRNAs such as H19 and MALAT1 are redox sensitive and influence glutathione synthesis, autophagy, and apoptosis. CircRNAs stabilize miRNAs and scaffold redox signaling complexes. Profiling non-coding RNAs via microarrays or RNA-seq provides a multilayered perspective on redox regulation.

### 6.4. Redox Metabolites as Exposure Biomarkers

Metabolomic analyses have demonstrated that environmental oxidative stress leads to distinct alterations in metabolic pathways. Among the most informative biomarkers are the ratios of reduced to oxidized glutathione (GSH/GSSG), malondialdehyde (MDA), and 4 hydroxynonenal (4 HNE), which collectively reflect the extent of lipid peroxidation and overall oxidative damage. The cysteine/cystine ratio serves as a sensitive indicator of the thiol–disulfide redox state, while the ratios of NADH/NAD^+^ and NADPH/NADP^+^ provide insights into cellular redox potential and metabolic resilience [162]. Chronic exposure to traffic-derived pollutants has been shown to disrupt glutathione metabolism and amino acid pathways involved in oxidative stress responses, reinforcing the utility of these metabolites as precise and sensitive biomarkers of environmental exposure [85]. Notably, redox metabolism exhibits sex-specific characteristics: in males, oxidative stress impairs fertility through disruptions in glutathione balance and increased lipid peroxidation, whereas in females, estrogen-mediated regulation of Nrf2 modulates redox homeostasis and reproductive outcomes [163,164]. Additionally, the natural decline in redox equilibrium with age enhances susceptibility to environmentally induced metabolic disturbances, underscoring the importance of these biomarkers across the lifespan.

### 6.5. Integrative Redox-Epigenetic Interactions

Epigenetic and metabolic systems are closely intertwined through redox-dependent mechanisms. Oxidative metabolites, including α-ketoglutarate and 2-hydroxyglutarate, influence TET and KDM enzyme activity, establishing a direct biochemical link between metabolism and chromatin regulation [91]. Multi-omics approaches combining methylome, transcriptome, and metabolome profiling have revealed pollutant-specific signatures. For instance, diesel exhaust exposure is associated with hypomethylation of *Nrf2*, increased oxidized glutathione, and miR-34a up-regulation, representing a composite fingerprint of environmental redox imbalance [85,165]. This integrative perspective not only reveals mechanisms of redox regulation but also enables the discovery of biomarkers for personalized risk assessment and therapeutic targeting.

### 6.6. Translational and Clinical Implications

Integration of epigenetic and metabolomic biomarkers has the potential to strengthen exposomics (the study of lifelong environmental exposures) by linking external exposures to internal biochemical responses and molecular changes that precede overt disease [166,167]. In principle, these profiles could support earlier exposure stratification and hypothesis-driven screening for pollutant-associated risk states; in practice, most reported signatures remain insufficiently standardized and validated for clinical decision-making. Key constraints include heterogeneous exposure assessment, confounding by smoking, diet, socioeconomic factors, comorbidities, and medication use, and substantial technical variability introduced by sample collection, storage, batch effects, and cross-platform differences.

Artificial intelligence–assisted multi-omics modeling can accelerate biomarker discovery by integrating high-dimensional features and correlating redox signatures with health outcomes [168,169,170]. However, model performance and interpretability are highly sensitive to cohort size, population diversity, missing data, and analytic choices, and overfitting remains a major risk when candidate features greatly outnumber participants. Accordingly, robust translation requires clearly defined context-of-use, rigorous internal and external validation, and assessment of whether a candidate biomarker adds actionable value beyond established clinical and exposure variables.

Beyond diagnostics, identifying redox-sensitive pathways may inform prevention and intervention strategies. Activation of NRF2 and SIRT1 pathways through nutraceuticals (e.g., sulforaphane, resveratrol, and related compounds) can modulate redox equilibrium and may reverse some stress-responsive transcriptional programs in experimental systems [171,172]. For human translation, however, the central need is prospective, well-controlled studies that couple exposure measurement with standardized biomarker assays and clinically meaningful endpoints.

## 7. Future Perspectives

The integration of redox biology with epigenetic and multi-omics profiling represents a paradigm shift in how we understand, measure, and intervene in oxidative stress–related diseases. Redox signaling, once considered a damaging byproduct of metabolism, is now appreciated as a dynamic regulator of gene expression, chromatin remodeling, and cellular fate decisions. Moving forward, the convergence of environmental health, redox epigenetics, and precision medicine will drive the discovery of new diagnostic tools and therapeutic strategies [173]. First, expanding longitudinal cohort studies with multi-omics measurements will help establish causal links between environmental exposures, oxidative stress, and chronic disease. Current studies often suffer from limited sample sizes, a cross-sectional design, and a lack of exposure quantification. Integrating redox biomarkers, DNA methylation profiles, and metabolic signatures into existing exposomic datasets will improve resolution and generalizability. High-resolution metabolomics, single-cell epigenomics, and spatial transcriptomics will allow the dissection of redox heterogeneity across tissues and cell types.

Second, the development of portable sensors and wearable technologies to monitor redox parameters in real-time may revolutionize exposure surveillance and risk assessment [174]. Biosensors that detect breath ethane, exhaled nitric oxide, or salivary 8-OHdG could provide non-invasive, dynamic indicators of oxidative load. Combining these sensors with artificial intelligence and machine learning will support personalized redox tracking.

Third, therapeutic innovation must target the root causes of redox imbalance. While antioxidant supplementation has yielded inconsistent results, targeting upstream regulators such as Nrf2, SIRT1, and AMPK may offer more durable benefits. Epigenetic drugs (DNMT inhibitors, HDAC inhibitors) and NAD^+^ boosters (nicotinamide mononucleotide, NR) are under investigation for age-related and metabolic diseases [175]. CRISPR-based epigenome editing and RNA therapeutics may enable precise modulation of redox-sensitive gene expression in the future.

Lastly, ethical and societal considerations surrounding multi-omics data integration, privacy, and equity must be addressed. Vulnerable populations disproportionately exposed to environmental oxidative stress must be prioritized in research and interventions. Ensuring equitable access to redox-targeted therapeutics and diagnostics will be essential for realizing the promise of redox precision medicine [176].

## 8. Conclusions

Oxidative stress is a plausible mechanistic bridge linking environmental exposures to aging phenotypes and chronic disease. Across major pollutant classes—including airborne particulates and gases, heavy metals, pesticides, engineered nanoparticles, and micro-/nanoplastics—multiple upstream insults converge on a limited set of ROS-generating and ROS-amplifying nodes, most notably mitochondrial dysfunction, NADPH oxidase activation, and redox cycling/Fenton chemistry. The resulting redox imbalance can damage lipids, proteins, and DNA, activate inflammatory signaling, and interface with epigenetic regulation (DNA methylation, histone modifications, and non-coding RNAs) in ways that could contribute to persistent gene-expression reprogramming and accelerated biological aging.

From a translational standpoint, the field is constrained less by a lack of candidate mechanisms than by the difficulty of measuring redox biology reliably in vivo. Established oxidative-damage markers and redox cofactors provide useful anchors, but they vary substantially in analytical specificity and biological interpretability, and no single marker is sufficient. Emerging epigenetic and multi-omics signatures offer promising approaches for characterizing exposure–response networks, yet most remain exploratory and require rigorous validation, standardization, and prospective testing before they can support clinical decisions. Future progress will therefore depend on integrating high-quality exposure assessment with fit-for-purpose biomarker panels, transparent analytic pipelines, and study designs that enable replication and causal inference.

## Figures and Tables

**Table 1:** Practical maturity tiers for oxidative stress biomarkers.

Tier	Tier Name	Definition/ Evidence Level	Representative Biomarkers	Typical Analytical Methods	Strengths	Limitations	Recommended Use Context
**Tier 1**	**Validated Clinical Biomarkers**	High analytical validity and reproducibility; strong human data; widely accepted as reliable indicators of oxidative stress in vivo	F_2_-isoprostanes, protein carbonyls, 8-OHdG (urine/plasma), AOPPs	GC-MS, LC-MS/MS, HPLC-ECD, validated ELISA	High specificity (especially MS-based), stability, cross-study comparability	Cost, technical expertise required; some variability across assays (ELISA vs. MS)	Clinical studies, epidemiology, intervention trials, translational research
**Tier 2**	**Established Research Biomarkers**	Widely used in experimental and clinical research; moderate specificity; good but variable standardization	MDA (TBARS), 4-HNE adducts, 3-nitrotyrosine, dityrosine, IMA	Spectrophotometry (TBARS), ELISA, HPLC, Western blot	Accessible, cost-effective, large historical dataset	Limited specificity (e.g., TBARS), pre-analytical variability, assay artifacts	Preclinical studies, cohort studies, comparative analyses
**Tier 3**	**Functional Redox Indicators**	Reflect cellular redox status or antioxidant capacity rather than direct oxidative damage	GSH/GSSG ratio, NAD^+^/NADH ratio, antioxidant enzyme activities (SOD, catalase, GPx), total ROS (DCFH-DA)	Enzymatic assays, fluorescence probes, LC-MS, spectrophotometry	Functional insight into redox balance; dynamic and responsive	Indirect measures; high sensitivity to sample handling; limited specificity for oxidative damage	Mechanistic studies, intervention monitoring, systems biology integration
**Tier 4**	**Mechanistic/Pathway-Linked Biomarkers**	Provide insight into specific oxidative pathways or molecular mechanisms; often tissue- or context-dependent	Oxidized LDL (oxLDL), mitochondrial ROS markers, ER stress markers (CHOP, GRP78), lipid aldehyde–protein adducts	Immunoassays, Western blot, targeted MS, imaging-based assays	Mechanistic specificity; links oxidative stress to disease pathways	Limited standardization; often not comparable across studies; context-dependent interpretation	Mechanistic studies, disease-specific investigations
**Tier 5**	**Emerging Epigenetic and Multi-Omics Biomarkers**	Early stage or rapidly evolving markers reflecting integrated redox effects on gene regulation and metabolism	DNA methylation (e.g., Nrf2, SOD2), histone marks (H3K27me3), miRNAs (miR-21, miR-34a), metabolomic redox signatures	Bisulfite sequencing, ChIP-seq, RNA-seq, LC-MS metabolomics	High dimensionality; captures long-term and systemic effects; potential for precision medicine	Limited validation; high variability; complex interpretation; lack of clinical standardization	Discovery research, exposomics, systems biology, AI-driven biomarker development
**Tier 6**	**Exploratory/Experimental Biomarkers**	Early proof-of-concept markers; limited validation; often technology-driven	Real-time ROS sensors, wearable biosensors (e.g., breath ethane, salivary 8-OHdG), novel nanoparticle-based probes	Biosensors, microfluidics, real-time imaging, electrochemical detection	Innovative, non-invasive potential; real-time monitoring	Limited reproducibility; lack of standardization; not yet clinically validated	Technology development, pilot studies, future translational pipelines

**Table 2:** Emerging epigenetic and metabolomic biomarkers of oxidative stress: mechanisms, analytical platforms, and translational potential.

Biomarker Class	Representative Markers	Mechanistic Insight	Typical Analytical Platforms	Maturity Level	Strengths	Key Limitations	Translational/ Research Use
**DNA methylation**	Hypomethylation of *Nrf2*, *SOD2*; hypermethylation of DNA repair genes	Reflects redox-driven disruption of methylation machinery (DNMT/TET imbalance); indicates epigenetic drift and impaired antioxidant responses	Bisulfite sequencing, methylation arrays, targeted PCR-based assays	Emerging (Tier 5)	Stable, cumulative exposure marker; tissue- and exposure-specific signatures	Cell-type specificity; variability across cohorts; limited clinical standardization	Exposure assessment, longitudinal cohort studies, early disease risk stratification
**Histone modifications**	↑H3K27me3, ↑H3K9me3; altered acetylation (H3/H4)	Chromatin remodeling under oxidative stress; repression of antioxidant and detoxification genes	ChIP-seq, ChIP-qPCR, mass spectrometry	Emerging (Tier 5)	Mechanistic specificity linking redox state to gene regulation	Technically demanding; low standardization; difficult clinical scalability	Mechanistic studies, pathway analysis, epigenetic drug targeting
**Non-coding RNAs (miRNA, lncRNA, circRNA)**	↑miR-21, ↑miR-34a, ↓miR-146a; MALAT1, HOTAIR	Post-transcriptional regulation of redox-sensitive pathways (Nrf2, SIRT1, inflammation)	RNA-seq, microarrays, qRT-PCR	Emerging (Tier 5)	Detectable in circulation; minimally invasive; responsive to environmental exposure	Context-dependent expression; normalization challenges; limited reproducibility across platforms	Non-invasive biomarker development, disease prediction, therapeutic targeting
**Redox metabolites**	↓GSH/GSSG ratio, ↑MDA, ↑4-HNE, ↓NAD^+^/NADH ratio	Reflects systemic redox imbalance, antioxidant depletion, and metabolic dysfunction	LC-MS/MS, NMR spectroscopy, enzymatic assays	Intermediate (Tier 3–5 bridge)	Functional and quantitative; integrates metabolic state with oxidative stress	High sensitivity to pre-analytical conditions; variability across tissues and assays	Systems biology integration, intervention monitoring, metabolic profiling
**Protein adducts/lipid-derived adducts**	4-HNE–protein conjugates, oxidized protein adducts	Indicate cumulative lipid peroxidation and protein damage; link oxidative stress to cellular dysfunction	ELISA, Western blot, targeted MS	Intermediate (Tier 2–4 bridge)	Mechanistic relevance; relatively stable compared to ROS	Limited specificity; assay variability; influenced by turnover rates	Disease-associated oxidative damage assessment, mechanistic validation
**Multi-omics signatures**	Integrated methylome–metabolome–transcriptome profiles	Captures systems-level response to oxidative stress and environmental exposure; identifies pollutant-specific signatures	Integrated omics platforms (LC-MS, RNA-seq, methylome sequencing), AI/ML modeling	Emerging (Tier 5–6)	High dimensionality; enables precision medicine and exposomics	High cost; complex data integration; limited standardization and validation	Exposomics, predictive modeling, personalized risk assessment
**Real-time/sensor-based biomarkers**	Breath ethane, exhaled NO, salivary 8-OHdG, wearable ROS sensors	Reflects dynamic, real-time oxidative processes and environmental exposure	Biosensors, electrochemical detection, microfluidics	Exploratory (Tier 6)	Non-invasive, real-time monitoring potential	Limited validation; calibration challenges; environmental interference	Pilot clinical monitoring, environmental exposure tracking, future digital health applications

Arrow up is: expression upregulated; arrow down is expression downregulated.

## Data Availability

No new data were created or analyzed in this study. Data sharing is not applicable to this article.

## Abbreviations

The following abbreviations are used in this manuscript:

4-HNE	4-hydroxynonenal
8-OHGua	8-hydroxyguanine
8-OHdG	8-hydroxy-2′-deoxyguanosine
ACB	albumin cobalt-binding
AMPK	AMP-activated protein kinase
AOPPs	advanced oxidation protein products
AP-1	activator protein-1
CHOP	C/EBP homologous protein
DCFH-DA	2′,7′-dichlorodihydrofluorescein diacetate
DNMTs	DNA methyltransferases
DNPH	2,4-dinitrophenylhydrazine
DRP1	dynamin-related protein 1
ER	endoplasmic reticulum
ESR	electron spin resonance
ETC	electron transport chain
GC-MS	gas chromatography–mass spectrometry
GSH	glutathione
GSSG	glutathione disulfide
HATs	histone acetyltransferases
HDACs	histone deacetylases
HPLC	high-performance liquid chromatography
HPLC-ECD	HPLC coupled with electrochemical detection
IDH	isocitrate dehydrogenase
IMA	ischemia-modified albumin
IsoPs	F_2_-isoprostanes
MAM	mitochondria-associated membrane
MDA	malondialdehyde
NAPDH	nicotinamide adenine dinucleotide phosphate
NMN	nicotinamide mononucleotide
NO_2_	nitrogen dioxide
NOX	NADPH oxidase
NR	nicotinamide riboside
NRF1	nuclear respiratory factor 1
NSCs	neural stem cells
O_3_	ozone
OPA1	optic atrophy protein 1
PAHs	polycyclic aromatic hydrocarbons
PFAS	per- and polyfluoroalkyl substances
PGC-1α	peroxisome proliferator-activated receptor gamma coactivator 1-alpha
POPs	persistent organic pollutants
ROS	reactive oxygen species
SAM	S-adenosyl-methionine
SASP	senescence-associated secretory phenotype
SOD	superoxide dismutase
TBARS	thiobarbituric acid reactive substances
TCA	tricarboxylic acid
TET	ten-eleven translocation
TLR	Toll-like receptor
UFPs	ultrafine particles
UPR	unfolded protein response
circRNAs	circular RNAs
lncRNAs	long non-coding RNAs
mPTP	mitochondrial permeability transition pore
miRNAs	microRNAs
mtDNA	mitochondrial DNA
mtROS	mitochondrial ROS
oxLDL	oxidized low-density lipoprotein
